# A comprehensive mobile application tool for disease surveillance, workforce management and supply chain management for Malaria Elimination Demonstration Project

**DOI:** 10.1186/s12936-021-03623-3

**Published:** 2021-02-16

**Authors:** Harsh Rajvanshi, Yashpal Jain, Nidhi Kaintura, Chaitanya Soni, Raja Chandramohan, Ramanathan Srinivasan, Vinay Telasey, Praveen K. Bharti, Deepak Jain, Mangeshi Surve, Sachin Saxena, Vilas Gangamwar, M. S. Anand, Altaf A. Lal

**Affiliations:** 1 Malaria Elimination Demonstration Project, Mandla, Madhya Pradesh India; 2 Information Technology, Sun Pharmaceutical Industries Ltd, Mumbai, Maharashtra India; 3 Swaas Systems Pvt Ltd, Chennai, Tamil Nadu India; 4 Indian Council of Medical Research-National Institute of Research in Tribal Health (ICMR-NIRTH), Jabalpur, Madhya Pradesh India; 5 Foundation for Disease Elimination and Control of India, Mumbai, Maharashtra India; 6 Present Address: Jphiego, Jaipur, Rajasthan India; 7 Present Address: Medtronic, Mumbai, Maharashtra India; 8 Present Address: RheinBrücke IT Consulting, Chennai, Tamil Nadu India

## Abstract

**Background:**

Health care technologies are now offering accountability, quality, robustness, and accuracy in disease surveillance and health care delivery programmes. With the advent of mobile hand-held devices, these technologies have become more accessible and adaptable for use by field staff working in remote areas. The Malaria Elimination Demonstration Project started collection of data and conduct of routine operations using paper-based reporting systems. Observing the need for a robust and quality digital mobile application, a comprehensive mobile application tool was developed that allowed the project to conduct disease surveillance, workforce management and supply chain management.

**Methods:**

In June 2017, the project conceptualized a comprehensive mobile application tool in the local language (Hindi) for disease surveillance, human resources management, and supply chain management. The tool is also available in English. Solution for Community Health-workers (SOCH) mobile app is an android native application developed using android SDK and web-based tool using MVC.net framework. Construction of the application started in November 2017 and rolled out its pilot in April 2018, followed by pan-district roll out in July 2018. The application uses self-validation tools to ensure high level of data quality and integrity.

**Results:**

The software is available in android based hand-held devices and web-screens with built-in data analytical capabilities. Using SOCH, the project has now successfully digitized its routine surveillance, attendance, tour plans, supply chain management components. The project has documented a reduction in 91% indigenous cases in the district, 60% improvement in stock accountability, and 99.6% accuracy in data collected through the mobile application.

**Conclusion:**

SOCH is an excellent and user-friendly tool, which can be customized for any public health management programme. The system ensures accountability and data robustness, which is needed for malaria elimination efforts throughout the country. The mobile application can be adapted for English or any other Indian or international language for use for malaria or any other disease surveillance and control programme. Another expansion feature of this mobile application is incorporation of indicators for Indoor Residual Spraying (IRS), Long-Lasting Insecticidal Nets (LLINs), and minor engineering by the residents of community under surveillance. The authors believe that it would be highly desirable and appropriate for an international organization, such as the World Health Organization (WHO), to conduct an independent comparison of all available mobile e-surveillance tools, so that a high-performing and globally suitable system can be selected for use in malaria elimination programmes. The Foundation of Disease Elimination and Controlof India has decided to make the SOCH mobile application available to anyone who would like to use it for disease surveillance and health care programmes.

## Background

Disease morbidity and mortality estimates are the cornerstone of effective implementation of any public health programme. Data on burden of malaria in India has been a topic of debate, with differences in data reported by national program and the World Health Organization (WHO) [1–3]. There is a consensus, however, that collection and reporting of data is influenced by faulty reporting and lack of technology and tools for real-time and un-editable primary data from the field. In this context, it is important to point out that the Sri Lanka eliminated malaria by extensive use of health-care technology using GIS [4].

With the explosion of mobile phone usage in India, there is an opportunity to use mobile e-surveillance and disease reporting systems. For instance, India has 1211.80 million telephone connections, out of which 1188.99 million (98.1%) are mobile phones, with almost all of them equipped with internet connections [1]. Moreover, the preferred operating system is Android, which offers extreme customizability for making user-friendly and safe mobile application tools. Despite such savviness of mobile phones in the country, public health data reporting is being done by national programmes through the cumbersome paper-based reporting formats. These formats are physically submitted to the respective data centres by the health staff and are subjected to inadequate data validation.

Malaria Elimination Demonstration Project is India’s first public–private partnership initiative between the Government of Madhya Pradesh, Indian Council of Medical Research, and Foundation for Disease Elimination and Control (FDEC) of India, which is a CSR initiative of Sun Pharmaceutical Industries Ltd. The goal of the MEDP was to demonstrate elimination of malaria from 1233 villages of district Mandla using robust surveillance, case management, and vector control. The findings of the MEDP are presented in the companion papers as a thematic series [2–8].

This paper describes development and use of mobile application tool, Solution for Community Health -orkers (SOCH), which allows surveillance, supply chain management, and workforce management. Some of the salient features of SOCH are: (1) Electronic surveillance and disease reporting systems, stock flow, attendance management, intra-project communication; (2) Enablement of Advance Tour Plans (ATPs) for the field staff along with ‘need to know’ access to systems based upon the work assigned by the project; (3) Global Positioning System enablement for staff along with unique IDs assigned to each individual and household of the district; (4) Built- in data validation protocols and uplink with server in real-time allowing the project reports to view anytime and anywhere; (5) Stock flow using indents and requisitions along with auto-deduction of stock at-hand while performing active case detection; and (6) Readily available Key Performance Indicators that can be pulled from the system in real-time.

SOCH system plugs the gap of data unification and integrity by maintaining a population database up-to the level of each individual in a district. This database is regularly updated, is confidential, ensures data integrity and can be used by initiatives even outside of health programmes.

## Methods

### General project area characteristics

Mandla is a district in the central state of Madhya Pradesh in Jabalpur division spread over an area of 8771 sq Kms. The district consists of nine development blocks, 281 sub centres and 1233 villages with approximately1.15 million population [5]. Mandla shares borders with four other districts of Madhya Pradesh and the state of Chattisgarh. MEDP covered the entire district through its field staff of 235 Village Malaria Workers and 25 Malaria Field Coordinators.

### Designing of the tool

The ‘SOCH’ mobile application was developed as an android native application using Android SDK with administration web site based on MVC.net Framework. The application integrated with Azure SQL Database with ASP.net Web API. Using REST API, the android application was independent from Web application which yielded higher performance and worked even without network connectivity in the field.

**Fig. 1:**
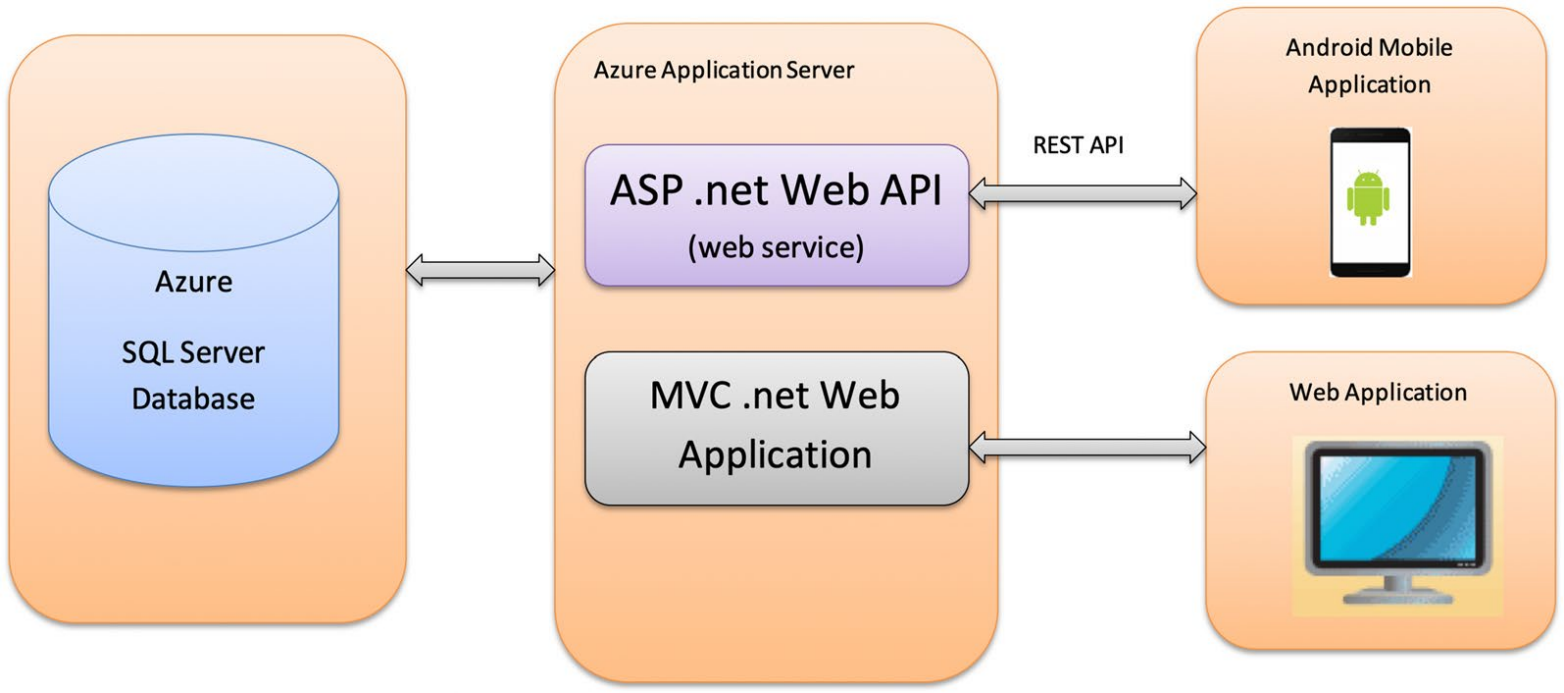
A schematic of the technical architecture of SOCH mobile application

The SOCH web application was used for administrative purposes consisting of Key Performance Reports. It was developed using Microsoft technologies—MVC ASP.net framework with C#. The web service was developed using ASP.net Web API and the android application was developed using Android SDK with Java. SQL Server database Server & Application server were hosted in Microsoft Azure (Fig. 1).

The tool was conceptualized along with the beginning of field operations in August 2017. The tool was based on the existing HiDoctor framework of SwaaS Systems. The customization to Mandla project required 15 person-weeks of programmer time. Focus was to make application light and efficient so that it could run on existing android-based handsets of all the front-line staff of the project. The application was made in the local language ‘Hindi’ as default with added option of switching to English as well.

### Form design

The forms were digitally designed to correspond the existing reporting systems of the project. Two different interfaces were considered—mobile version for primary users and desktop version for the district-level staff. These forms were modified versions of the forms used by the National Vector Borne Disease Control Programme. These included HouseholdInformation form, Active Case Detection form, Passive Data Collection forms, Indoor Residual Spraying (IRS) monitoring checklists, Long-Lasting Insecticidal Nets (LLINs) monitoring checklist, Mass Screening and Treatment form, Community Camp Case detection form, Stock Inward Request form, Stock Acknowledgement receipts, Leave Applications, and Advance Tour Plan (ATP) formats.

The risk of error was minimized by auto-population and self-validation of maximum fields in line-list of fever and malaria cases using the household census information that was already uploaded in the database. To reduce risk of error in stock calculation, auto deduction protocols were designed with logics of different scenarios a user may encounter. The forms were designed in local language with user friendly interface involving contrasting colours, big fonts, and complimenting the natural finger movement of user while working on a touch-screen phone.

**Fig. 2:**
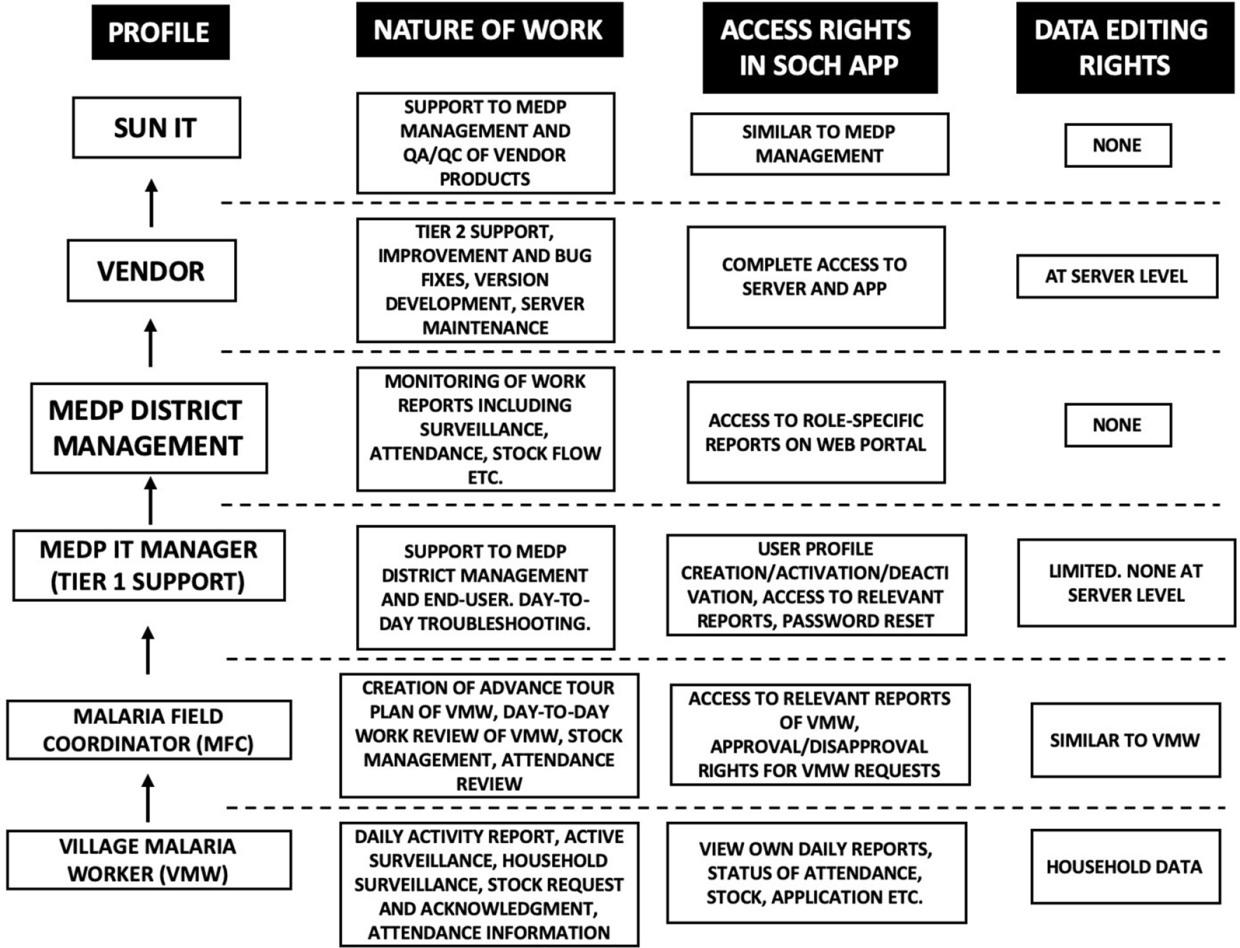
A schematic of different users of SOCH mobile application along with their access rights

The desktop version forms were similar to those of mobile version. The purpose was that the data entry operators could enter the data manually into the system during a breakdown of network or mobile phone. Desktop version also featured an output interface for the project management to pull-out key performance reports. A schematic of different users of SOCH along with their access rights can be visualized from Fig. 2.

### Workflow

The workflows of the mobile application along with one time and recurring costs is given in Table 1. Each user filled the forms each day and uploaded a Daily Activity Report (DAR) to the server. If there was absence of net connectivity, the DAR could be kept in ‘draft’ mode and uploaded to the server within a time period of 3 days—following which the application went in a ‘lock-down’ mode and refused to move forward (at user-level) unless the IT team was notified and systems were reset. This functionality helped the management in ensuring that the data was received on-time. Once the DAR was uploaded to the server, it was readily available on the reports platform at district level. The DAR consisted of any activity done during the day, including active case detection, passive data collection, stock flow activities, and attendance requests.

**Table 1:** Cost of deploying SOCH mobile application in Mandla district

Project item description	Cost of unit (USD)	Frequency	Total cost
A. Per user charges for using the mobile application (260 users)	$0.69	Per month	$181
B. Hosting and software infrastructure charges	$174	Per month	$174
C. Total technical development cost	$16,444.12	One time	$16,444
D. Training (includes trainers, training materials, transport, meals, allowances) for entire district. Two trainings—pilot and pan-district roll out	$8,621.92	One time	$8622
Total (C + D)—one time costs			$25,066
Total (A + B)—annual recurring costs			$4260

### The SOCH dashboard

**Fig. 3:**
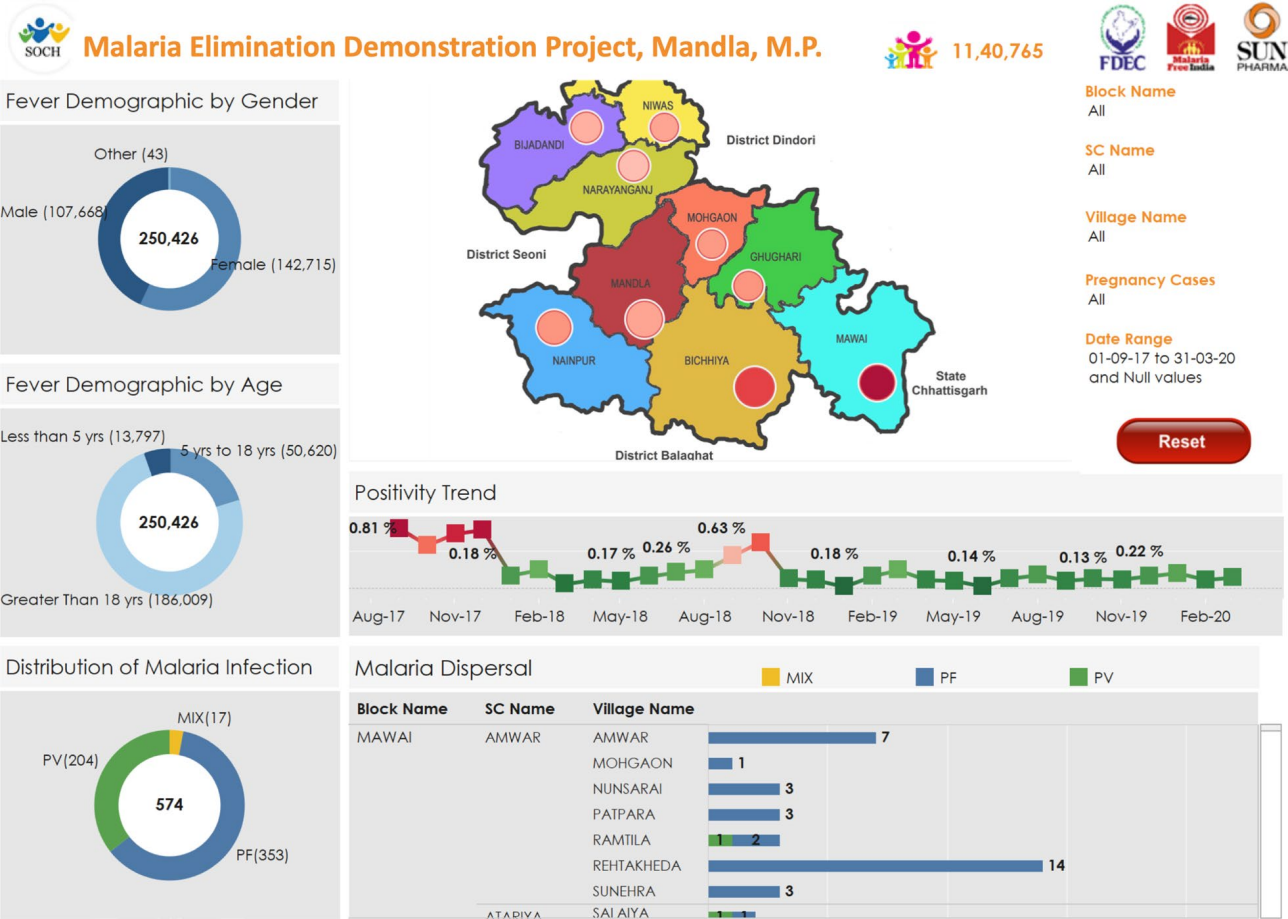
Screenshot of the visual dashboard of the SOCH mobile application data for the Malaria Elimination Demonstration Project

This tool was designed and developed to provide the visualization of malaria prevalence in the project area. The user could slice and dice the data as per gender, age-groups, block, sub-centre, villages, and pregnancy cases. It also showed the malaria month-wise trend (Fig. 3).

### Approach to dashboard development

Data transformations rules were created using Informatica ETL. Data was extracted from SOCH system, translated (as required for dashboard) and loaded into Oracle database. It loaded fever and malaria cases—surveyed/treated for population on daily basis. Graphical user interface, called as Dashboard, is designed using 'Tableau' Visualisation Tool. Tableau picked up the data from Oracle Database and displayed current analysis.

#### Pilot run

Once the technical development of the mobile application was completed, a pilot-run was carried in two out of nine blocks of the district. The four-day training and pilot roll-out happened in May 2018. The agenda of the pilot plan consisted of training of core office staff, data entry operators, and field supervisors on day 1. Day 2 involved full day sessions to train the users in the presence of their respective field supervisors. Day 3 consisted of mop-up sessions and Q&A for the entire team. Printed user manuals in local language were circulated to all the trainees in-advance. After the training, the two blocks tested the application for 15 days in their respective work areas. Comprehensive feedbacks were obtained and discussed with the entire team.

### Pan-district rollout

Based upon the recommendations from the pilot, several changes were made in the mobile application resulting in the version 2.0 of SOCH. Key recommendations following the pilot consisted of the application crashing while performing certain functions, missing household data, problems in hierarchies of blocks and villages, and additional monitoring reports requested by the field supervisors. Pan-district roll-out and training was conducted in July 2018 for the entire staff of the project.

### Attendance and stock management

The application used a calendar-based interface where the user could view their attendance, apply for leaves, and see the work-done at any particular date. The automated attendance system was based on user-hierarchies for approval/disapproval of leaves. Similarly, the automated stock management system enabled the user to raise indents based upon their existing stock availability and projected requirements in the upcoming months. The stock requests had a cut-off date of 24th of every month. The requests were approved by their immediate supervisor and approved by the Supply Chain Officer and District Officer at district-level. Following this, the physical stock was allocated to the users which was collected by their supervisors during the monthly meeting on 2nd of every month. The supervisors handed-over the physical stock to their workers and an acknowledgement of stock-received was sent by the workers directly to the district-level.

## Results

### Programme set up

The time from conceptualization till the implementation of the application took about 10 months. The planning started in September 2017 and pan-district roll out was completed in July 2018. The one time cost incurred was $25,066 with monthly recurring expenses of $355 maintaining an average user database of 260 users. During the paper-based reporting system, an average monthly cost of $1200 was borne owing to the cost of printing, transport, and digitization of the paper forms.

### Compliance

**Fig. 4:**
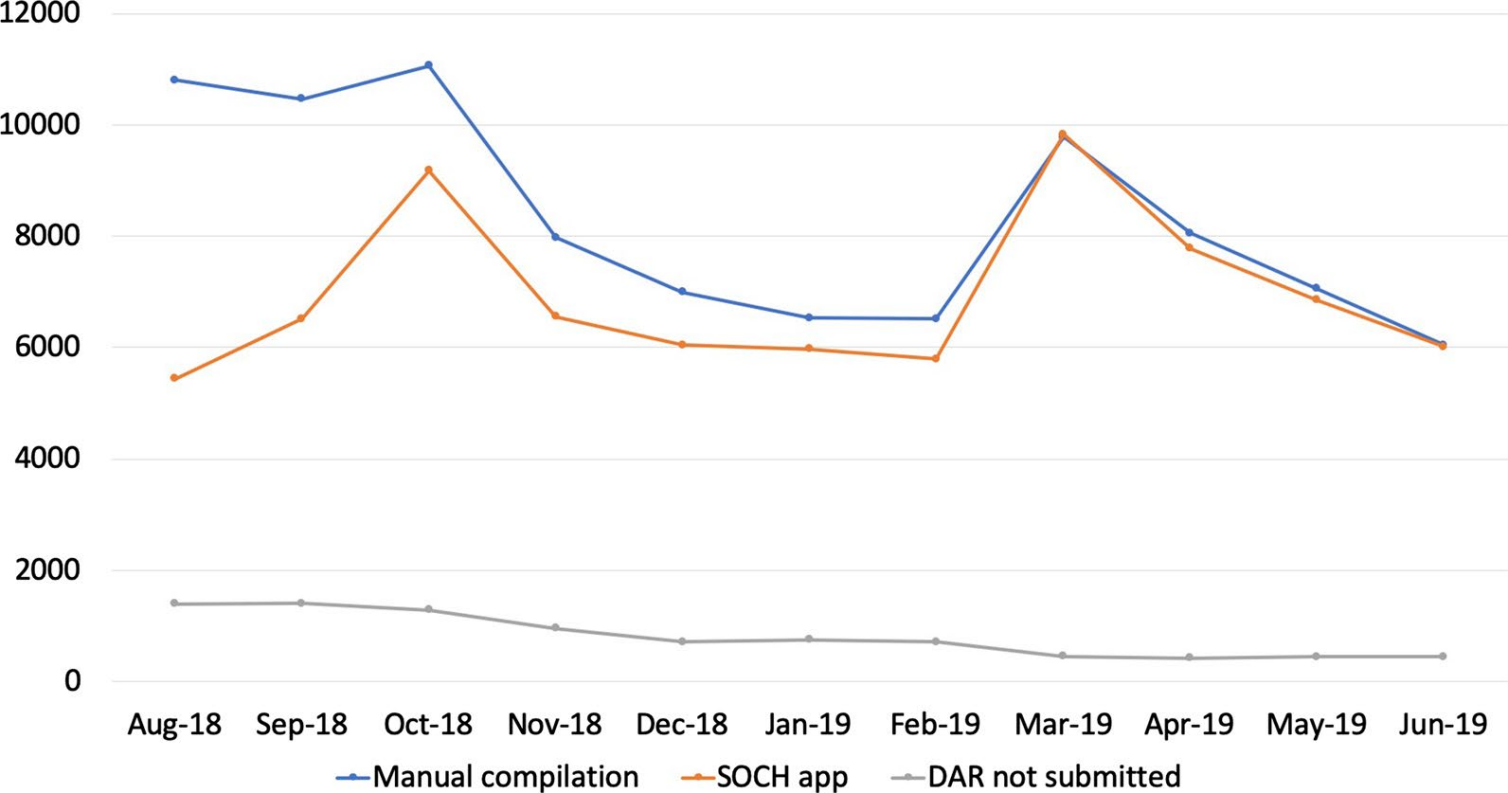
The difference in reporting of febrile cases by paper-based reporting (blue line) and SOCH mobile application (orange line) from introduction of the digital system in August 2018 to June 2019. The grey line indicates the number of Daily Activity Reports (DAR) not submitted over the same time period

The roll-out was completed in July 2018. The process of weaning off from paper based reporting systems took significant amount of time (Fig. 4). Initially, the electronic report from SOCH and manual data (both mutually exclusive), were consolidated the back-end due to sub-par compliance of SOCH. In August 2018, there was a difference in fever surveillance data by 49.6% between both the systems. With regular troubleshooting, the difference reduced to 17% in October 2018 and 8% in January 2019. After further gap analysis and efforts to eliminate paper based reporting systems, the system stabilized at 0.4% difference in March 2019. Similarly the daily activity reports (DAR) not submitted in August 2018 was at 21.5% (1399) and stabilized at 7% (455) in March 2019 (grey line). In an ideal scenario, the total DAR to be uploaded in a month would be 6500, assuming 260 users and 25 working days. The number in the tune of 455 missed DARs would attribute to public holidays, users on-leave, and vacant positions.

### Key indicators and project performance

**Fig. 5:**
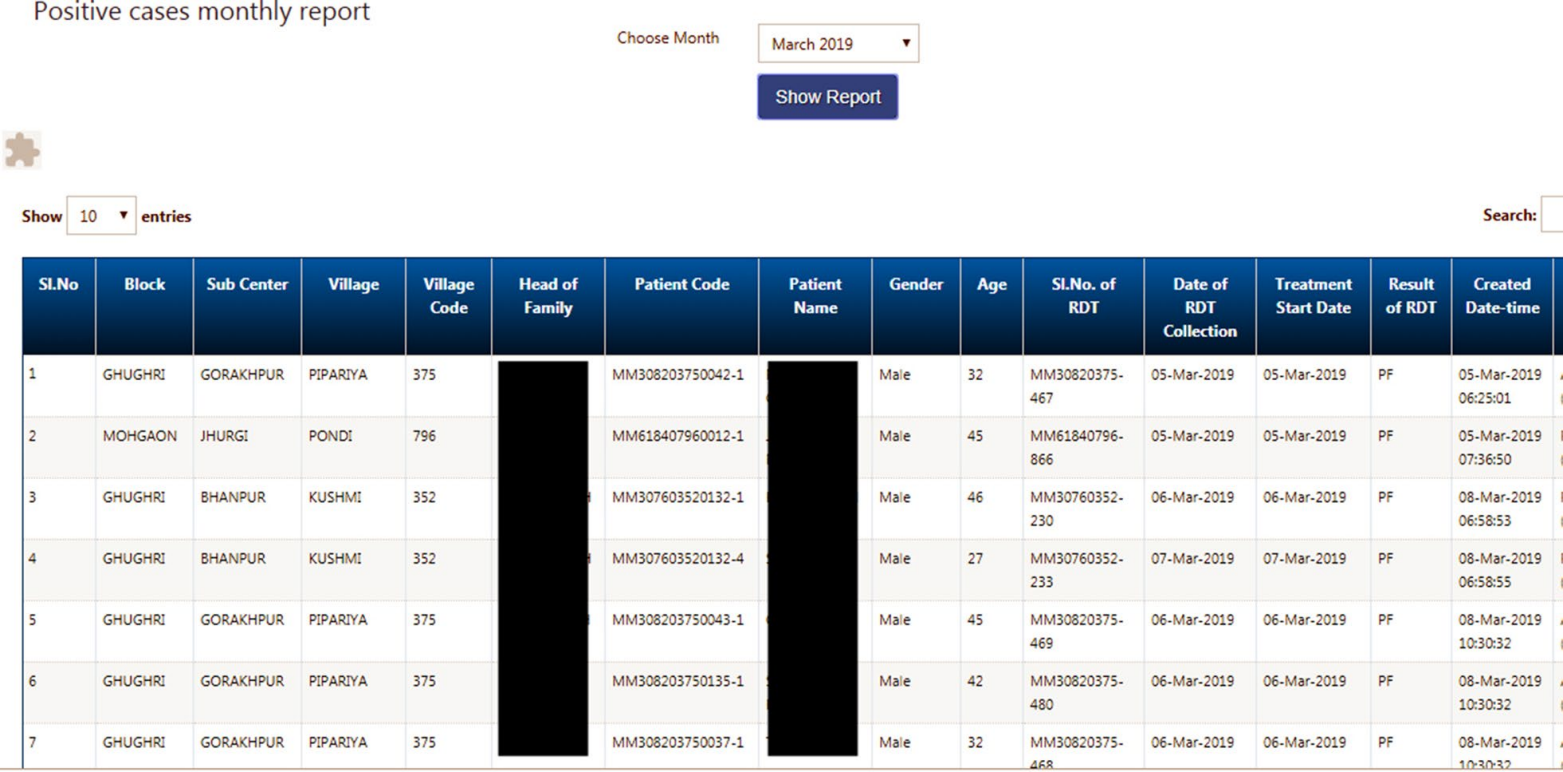
Screenshot of the positive cases line-list as seen in the desktop reports of the SOCH mobile application

Robust data allowed the project to curate its strategies to meet the specific objectives. Before SOCH, it took 40 days (30 days of the month + 10 days data compilation time) to develop monthly surveillance reports. After implementation of the application, the reports were available in real-time with a maximum possible delay of three days. The application did not allow the user to store the data in offline mode for more than three days. The overall indigenous cases reduction by 91% was an outcome of the data reporting systems put in place using SOCH [2]. After introduction of the automated supply chain management system, stock accountability went up by 60%. Adherence to Advance Tour Plans (ATPs) went up-to 95% from 62%. The load of hardcopy leave applications went down to a single screen format which allowed quick processing saving up-to 90% of the time of the manager. There was also a significant increase in target households covered by saving time from manual line listing of each fever and malaria positive patient (Fig. 5). The mobile application offered updated household-level data in real time [5].

## Discussion

The SOCH application is a good example of IT-based disease surveillance, which allowed precision and integrity. The application successfully digitized all operations of the Malaria Elimination Demonstration Project and left behind the paper based reporting systems only for exigencies. The framework is customizable and has potential to be scaled up in the entire state and country. It can be tailored for other public health programmes. A cost-analysis of paper-based surveillance systems vs. app-based surveillance data revealed the latter to be highly competitive and efficient tool. These features made SOCH and similar technologies as the new gold standard in disease surveillance and health care management.

The Government of India uses the Health Management Information System (HMIS) to analyse the national health data collected from various districts. The challenge of poor data quality is faced at national level [9]. The key contributing factor would be gaps at the level of data collection (paper-based system) and capacity to analyse and correct the data at district-level. SOCH plugs this gap by ground-level reporting into the system with capabilities using its pre-loaded population data base in the software and auto-generated KPI reports for the district-level administration.

A review of the mobile phone systems based health projects in Africa highlighted the need for accessibility, strong stakeholder collaboration, and adaptation to local contexts as chief contributors in success of any health technology tool. These projects have the potential to solve challenges of stock management, poor surveillance, and reporting systems [10–13]. SOCH has provided utility to meet these challenges in a real-field setting.

Any system that is developed by humans contains inherent errors and bugs which need fixing on a regular basis. At policy level, it might be felt that healthcare information technology (HIT) is a quick fix, but on the contrary it is the exact opposite. HIT is a long term solution requiring several revisions before the system can be considered ready for action [14].

Within a span of 15 months of the release of pilot, four versions of SOCH with several bug fixes and system revisions were released. During the process of achieving a state of equilibrium in the system, neo-luddism was observed. Neo-Luddism is a philosophy which opposes modern technology. It was mostly due to this philosophy that it took time achieve 99% reduction in physical and digital data discrepancy in the district. It was imperative to differentiate that the problems into ‘device’ problems or ‘human’ problems. Constant motivation and troubleshooting helped in achieving the objectives.

Similar attempt to improve data reporting was done in Uganda using SMS based software and achieved promising results. The positive shift in data reporting compliance was noted up-to 88.6%. Significant improvement was seen in stock management and prevention of stock-outs in the target districts [15]. SMS based reporting systems have been tested extensively in African subcontinent and yielded positive results [16–18]. Using SOCH, a reduction in stock-out situations by up-to 95% within six months of the application roll-out was observed. Accountability was achieved using the digital indents, acknowledgements, and auto-deduction of stock as the user performed fever surveillance in the villages.

Apart from the listed functions of SOCH in malaria surveillance, reporting, stock, and attendance management; the application also has the ability to capture the Indoor Residual Spray (IRS) operations and post-distribution usage of Long-Lasting Insecticidal Nets (LLINs) using comprehensive monitoring checklists built in the system. In South Africa, a mobile application dedicated to monitor the IRS was created and focussed on reducing the human exposure to insecticide sprayed areas [19]. It should be noted that exhaustive documentation of m-health projects is needed to replicate them on a broader level. In Africa, there are several sincere and successful m-health projects localized in specific areas. Lack of documentation and life cycle analysis is a road-block in escalating them to larger context [20].

India has launched a country-wide novel mobile application by the name of ANMOL (ANM On-Line) for the Auxiliary Nurse Midwives (ANMs). The android based application has been developed in collaboration with UNICEF and tablets have been provided to all the ANMs. The application features a digitized database of new-borns, pregnant women, and mothers in the area along with integrated workflow module for each ANM. The application also contains videos and images for IEC/BCC, and similar to SOCH, works in offline mode and synchronizes with the server as soon the internet is available. ANMOL is primarily limited to maternal and child health including immunization and family planning activities. Integration of SOCH and ANMOL may result in a more comprehensive health tool.

The entire population database, attendance management, and supply chain management tools of SOCH can be useful by integrating into ANMOL [21]. A comprehensive Integrated Health Information Programme (IHIP) has been launched by the Government of India to create standard compliant Electronic Health Records (EHR) of each citizen of India. The project has been started in Odisha and is in its pilot testing stages.

Management of supply chain is a challenge in Indian public health programmes. The application ensured the first-in-first-out (FIFO) system. It is difficult to expect from the ground-level staff to ensure timely utilization of near-expiry medicines. After implementing SOCH, there were zero incidences of failed FIFO from ground to district-level because the application kept a record of all expiry dates and rendered prescription for the patient by identifying the medicines from desired batch numbers and their expiry dates.

The SOCH had a few limitations as well. It had built-in GPS capabilities that could capture the location only when user registered his/her attendance. A continuously running GPS would have decreased the phone battery life. Additionally, SOCH was solely dedicated to MEDP. The data generated by the state government was manually added in the final reports. The manually added datadid not have same granularity as compared to the SOCH data. The data from sentinel surveillance sites including the private practitioners was included manually due to low-compliance from these sites.

The scope of the software can be expanded to include a wider range of diagnostics apart from Rapid Diagnostic Kits by including results of microscopy and PCR. Another expansion feature of this mobile app was incorporation of input indicators for the use of IRS, LLINs, and minor engineering by the residents of community under surveillance.

The Foundation of Disease Elimination and Control, India, has made the decision to make the SOCH mobile application available to any organization in and outside India, who would like to use it for disease surveillance and health care programmes. The authors believe that it would be highly desirable and appropriate for a third party organization, such as the WHO, to conduct a comparative study of available mobile e-surveillance tools, so that a high-performing and globally suitable system can be selected for use in malaria elimination programmes globally. If this is done in a timely manner, the collection and sharing of data can be better monitored at sub-national, national, regional and global levels.

## Data Availability

We have reported all the findings in this manuscript. The hardcopy data is stored at MEDP Office in Mandla, Madhya Pradesh and Indian Council of Medical Research-National Institute of Research in Tribal Health (ICMR-NIRTH), Jabalpur, Madhya Pradesh. Softcopy data is available on the project server of MEDP hosted by Microsoft Azure. If anyone wants to review or use the data, they should contact: Dr. Altaf A. Lal: Project Director – Malaria Elimination Demonstration Project, Mandla; Foundation for Disease Elimination and Control of India, Mumbai, India 482003; E mail: altaf.lal@sunpharma.com, altaf.lal@gmail.com.

## Abbreviations

ANMOL: ANM On-Line
ANM: Auxiliary Nurse Midwife
ATP: Advance Tour Plan
DAR: Daily Activity Report
EHR: Electronic Health Record
FDEC: Foundation for Disease Elimination and Control of India
FIFO: First-In-First-Out
HIT: Healthcare Information Technology
HMIS: Health Management Information System
IHIP: Integrated Health Information Programme
IRS: Indoor Residual Spraying
LLIN: Long-Lasting Insecticidal Nets
SOCH: Solution for Community Health Workers
WHO: World Health Organization
